# Astrocytes Protect Neurons against Methylmercury via ATP/P2Y_1_ Receptor-Mediated Pathways in Astrocytes

**DOI:** 10.1371/journal.pone.0057898

**Published:** 2013-02-28

**Authors:** Yusuke Noguchi, Youichi Shinozaki, Kayoko Fujishita, Keisuke Shibata, Yoshio Imura, Yosuke Morizawa, Christian Gachet, Schuichi Koizumi

**Affiliations:** 1 Department of Neuropharmacology, Interdisciplinary Graduate School of Medicine and Engineering, University of Yamanashi, Yamanashi, Japan; 2 Japan Science and Technology Agency, Core Research for Evolutional Science and Technology, Tokyo, Japan; 3 UMR_S949 INSERM, Université de Strasbourg, Etablissement Français du Sang-Alsace, Strasbourg, France; Albany Medical College, United States of America

## Abstract

Methylmercury (MeHg) is a well known environmental pollutant that induces serious neuronal damage. Although MeHg readily crosses the blood-brain barrier, and should affect both neurons and glial cells, how it affects glia or neuron-to-glia interactions has received only limited attention. Here, we report that MeHg triggers ATP/P2Y_1_ receptor signals in astrocytes, thereby protecting neurons against MeHg via interleukin-6 (IL-6)-mediated pathways. MeHg increased several mRNAs in astrocytes, among which IL-6 was the highest. For this, ATP/P2Y_1_ receptor-mediated mechanisms were required because the IL-6 production was (i) inhibited by a P2Y_1_ receptor antagonist, MRS2179, (ii) abolished in astrocytes obtained from P2Y_1_ receptor-knockout mice, and (iii) mimicked by exogenously applied ATP. In addition, (iv) MeHg released ATP by exocytosis from astrocytes. As for the intracellular mechanisms responsible for IL-6 production, p38 MAP kinase was involved. MeHg-treated astrocyte-conditioned medium (ACM) showed neuro-protective effects against MeHg, which was blocked by anti-IL-6 antibody and was mimicked by the application of recombinant IL-6. As for the mechanism of neuro-protection by IL-6, an adenosine A_1_ receptor-mediated pathway in neurons seems to be involved. Taken together, when astrocytes sense MeHg, they release ATP that autostimulates P2Y_1_ receptors to upregulate IL-6, thereby leading to A_1_ receptor-mediated neuro-protection against MeHg.

## Introduction

Methylmercury (MeHg), a well-known environmental pollutant, easily crosses the blood-brain barrier [1], [2] inducing several types of serious neuronal damage and disorders [3], [4], [5], [6]. Although most studies about MeHg-induced toxicity in the CNS have focused on its effects on neurons, MeHg, acting on a much higher number of glial cells, should affect their functions and viabilities. This is of great importance because it has become apparent that glial cells regulate a large variety of neuronal functions both in physiological and pathophysiological CNS [7]. However, the effects of MeHg on glial cells or neuron-to-glia interactions have received only limited attention.

Recently, it has become apparent that MeHg causes diverse responses in glial cells, i.e., it upregulates antioxidant genes [8], [9], while it rather inhibits the uptake of cysteine, a critical precursor of glutathione synthesis, leading to a decrease in antioxidants [10]. As one of the mechanisms of MeHg-induced neuronal loss is oxidative stress [11], [12], [13], [14], these glial responses by MeHg may greatly affect neuronal functions or viability. Inflammatory responses in glial cells are also involved in several types of neuronal damage. It has been reported that MeHg produces proinflammatory cytokines including interleukin-6 (IL-6) in glial cells [15], [16], [17]. In general, these cytokines facilitate inflammatory responses, leading to deterioration of the neuronal viability. However, we [18] and others [19] have already demonstrated that astrocytic IL-6 in response to various chemicals or insults protected neurons against oxidative neuronal death. However, the physiological or pathophysiological significance of the increased IL-6 in response to MeHg remains largely unknown, and even less is known about the mechanisms underlying MeHg-induced IL-6 in astrocytes.

Here, we demonstrate that MeHg upregulates several genes in astrocytes, among which IL-6 is the highest. And, as mentioned above, astrocytes protect neurons against MeHg by IL-6-mediated mechanisms. We also demonstrate that, when astrocytes sense MeHg, they release ATP that autostimulates P2Y_1_ receptors in astrocytes, thereby leading to IL-6 production via p38-mediated mechanisms. The released IL-6 appears to exhibit neuro-protection by upregulating adenosine A_1_ receptors in neurons.

## Materials and Methods

### Chemicals and Antibodies

Reagents were obtained from the following sources. Adenosine 5′-triphosphate (ATP), apyrase (grade III), bovine serum albumin (BSA), DPCPX, methylmercury (MeHg), MRS2179, (NH_4_)_2_S, Pb(NO_3_)_2_, suramin and Tris-maleate were purchased from Sigma Chemical (MO, USA). PD98059, SB203580, and SP600125 were purchased from Tocris bioscience (Bristol, UK). Recombinant rat IL-6 and anti IL-6 antibody were purchased from R&D Systems (MN, USA). Fura 2-acetoxymethyl ester (fura 2-AM) was purchased from Invitrogen (CA, USA). Polyclonal antibodies against total p38 and phosphorylated p38 were purchased from Cell Signaling Technology (MA, USA). Anti-MAP2 antibody was obtained from Chemicon (CA, USA). Anti-GFAP antibody was obtained from Millipore (MA, USA). Dextran T250 was purchased from Extrasynthase (Genay, France).

### Cell Culture

All of the animals used in this study were obtained, housed, cared for and used in accordance with the guidelines of the University of Yamanashi. Every effort was made to minimize the number of experimental animals used and their suffering. The culture of cortical neurons was prepared as described [20] with minor modifications. In brief, cerebral cortices dissected from 17-day-old fetal Wistar rats were digested with papain (9 units/ml) dissolved in PBS containing 0.02% L-cysteine monohydrate, 0.5% glucose, and 0.02% BSA at 37°C for 15 min. After enzyme treatment, cells were plated on BD PureCoat Amine 96 well cell culture plates (Becton, Dickinson and Company, NJ, USA) at a density of 8×10^4^ cells/well. The cells were maintained in DMEM supplemented with 1 mM glutamine, N1 supplement, 10 units/ml penicillin, and 10 µg/ml streptomycin under 5% CO_2_ at 37°C. The culture of cortical astrocytes was prepared as previously reported [20]. Cerebral cortices dissected from newborn Wistar rats were digested 0.1% Trypsin-EDTA at 37°C for 10 min. After enzyme treatment, the cells were dispersed by agitation through a pipette and plated in a flask. For purification of the astrocytes from the cortical cultures, the flask was shaken for 24 hr 7–10 days after seeding to remove detached cells. Then, astrocytes were subcultured in 6-well cell culture plates at a density of 2×10^5^ cells/well, 96-well cell culture plates at a density of 7×10^3^ cells/well, and LAB-TEK II chambered coverglass (Nalge Nunc International, NY, USA) at a density of 2.5×10^4^ cells/well.

### Mice

C57BL/6 mice (17-day-old fetal) were purchased from Japan SLC. P2Y_1_ knock-out mice (C57BL/6 background) have been developed as previously reported [21]. The cortical astrocytes from these mice were prepared as it is for the rat cortical astrocytes.

### WST-1 Assay

Neuronal viability and astrocytic viability were estimated by WST-1 assay using a cell counting kit (Dojindo, Kumamoto, Japan). After incubation with MeHg for 20 or 44 hr, 1/10 volume of WST-1 solution was added to the cell culture medium and incubated for an additional 4 hr. The absorbance of supernatants was measured with a microplate reader at 450 nm as the test wavelength and at 630 nm as the reference wavelength.

### DNA Microarray Analysis

For this experiment, astrocytes were exposed to 10 µM of MeHg for 2 hr. Converting total RNA (100 ng) to the targets for Affymetrix GeneChip DNA microarray hybridization was done according to the manufacturer’s instructions. The targets were hybridized onto a rat genome U34A GeneChip DNA microarray (Affymetrix, Santa Clara, CA) for 16–24 hr at 45°C. After hybridization, DNA microarrays were washed and stained on a Fluidics Station according to the protocol provided by Affymetrix. Afterward, the DNA microarrays were scanned, and then the images obtained were analyzed by GeneChip Operating System software (version 1.4; Affymetrix). The microarray data is available upon request.

### Quantitative RT-PCR

Total RNA was isolated and purified from astrocytes and neurons using RNeasy (Qiagen, Hilden, Germany) according to the manufacturer's instructions. Reverse transcription (RT)-PCR was performed using a one step primescript® RT-PCR Kit (Takara Bio Inc., Shiga, Japan) according to the manufacturer's protocol. The reaction mix contained 40 ng of total RNA, 200 nM primers, 100 nM TaqMan probe, TAKARA EX Taq*®* HS and PrimeScript™ RT enzyme Mix. RT-PCR amplification and real-time detection were performed using an Applied Biosystems 7500 Real-Time PCR System (Applied Biosystems, CA, USA). The reverse transcription was performed at 42°C for 5 min followed by inactivation at 95°C for 10 s. The temperature profile consisted of 40 cycles of denaturation at 95°C for 5 s, and annealing/extension at 60°C for 34 s. The sequence of the primers and probe for rat IL-6 were as follows: the TaqMan probe, 5′-CAGAATTGCCATTGCACAACTCTTTTCTCA-3′; the forward primer, 5′-CAGTGCATCATCGCTGTTCA-3′; and the reverse primer, 5′-CATATGTTCTCAGGGAGATCTTGGA-3′. The sequence of the primers and probe for rodent A_1_ receptor were as follows: the TaqMan probe, 5′-CGAGTCAAGATCCCTCTCCGGTACAAGA-3′; the forward primer, 5′-TCATCCTCACCCAGAGCTCC-3′; and the reverse primer, 5′-ATGGGTGTCAGGCCTACCAC-3′. Primers and the Taquman probe for GAPDH were obtained from Rodent GAPDH Control Reagents (Applied Biosystems). Mouse IL-6 expression was estimated using the probe set (Mm0046190-m1) from Applied Biosystems (Foster City, CA).

### Enzyme-linked Immunosorbent Assay of IL-6

The MeHg-induced IL-6 production from astrocytes was measured using a Quantikine® rat IL-6 immunoassay kit (R&D Systems, MN, USA). Astrocytes were incubated with MeHg (1 or 3 µM) in serum-free medium for 12 or 24 hr and the supernatants were collected. The assay was performed according to the manufacturer’s instructions. All standards and samples were measured with a microplate reader at a wavelength of 450 nm.

### Ca^2+^-imaging

Changes in intracellular Ca^2+^ were measured by the fura 2 method with minor modifications [22]. In brief, the culture medium was replaced with balanced salt solution (BSS) of the following composition (in mM): NaCl 150, KCl 5.0, CaCl_2_ 1.8, MgCl_2_ 1.2, HEPES 25, and d-glucose 10 (pH 7.4). Cells were loaded with fura 2 by incubation with 10 µM fura 2-acetoxymethyl ester (fura 2-AM) at room temperature (RT) in BSS for 45 min. After loading, the samples were mounted on a microscope (ECLIPSE TE2000-U, Nikon,Tokyo, Japan) equipped with a 75-W xenon lamp and band-pass filters of 340 and 380 nm wavelengths for measurement of the Ca^2+^-dependent signals (F340 and F380 nm). Image data were recorded by a CCD camera (ORCA-ER, Hamamatsu Photonics, Shizuoka, Japan). For evaluation, we used the ratio of F340/F380.

### Immunocytochemistry

Cells were fixed with 4% paraformaldehyde for 30 min at RT and they were incubated with the primary antibodies (anti-GFAP antibody at 1∶2000; and anti-MAP2 antibody at 1∶500) in a Can Get Signal A (TOYOBO, Osaka, Japan) for 24 hr at 4°C. Then, the cells were further incubated with Alexa 488- or Alexa 546-conjugated second antibodies (1∶2000) for 1 hr at RT. Fluorescent images were obtained by a laser scanning confocal microscope FV-1000 (Olympus, Tokyo, Japan).

### Measurement of Extracellular ATP

The extracellular ATP concentration of the MeHg-treated astrocytes was determined with an ATP bioluminescence assay kit CLS II (Roche Applied Science, Mannheim, Germany). Astrocytes were incubated with MeHg (1 or 3 µM) in serum-free medium and the supernatants were collected and boiled at 95°C for 10 min. Equal volumes of luciferin/luciferase reagents and samples (100 µl each) were mixed a few times by gentle pipetting. All standards and samples were measured with a Lumat LB9501 tube luminometer (Berthold, Wildbad, Germany). The ATP concentrations were calculated from the intensities of a series of standard ATP.

### Western Blotting

Cells were lysed and the lysates were electrophoresed with 10% SDS-PAGE gels and transferred to PVDF membranes. The membranes were blocked for 1 hr in Tris-buffered saline containing 0.1% Tween-20 and 5% BSA at RT and were incubated with primary antibodies (1∶5000) over night at 4°C. Membranes were then incubated with horseradish peroxidase-conjugated 2nd antibodies (1∶20000) for 1 hr at RT. Protein bands were visualized by rinsing the membrane with supersignal west pico chemiluminescence substrate (Thermo scientific, PA, USA). Images were obtained using LAS-4000 (Fujifilm, Tokyo, Japan).

### Enzyme Histochemistry

Enzyme histochemistry for ecto-ATPases activity has been performed on the basis of a previous report [23]. Briefly, cells were fixed with 4% paraformaldehyde for 30 min at RT and preincubated for 30 min at RT with Tris-maleate-sucrose buffer (250 mM sucrose, 50 mM Tris-maleate, pH 7.4) containing 2 mM CaCl_2_. The enzyme reaction was performed in a reaction buffer (2 mM Pb(NO_3_)_2_, 5 mM MnCl_2_, 2 mM CaCl_2_, 50 mM Tris-maleate (pH7.4), 250 mM sucrose, 3% dextran T250) with 1 mM of ATP as a substrate. After 1 hr reaction at RT, cells were washed with H_2_O and the ecto-ATPase activity was visualized by 0.5% (v/v) of (NH_4_)_2_S.

### Statistics

Data were expressed as means ± SEM. Student’s *t*-test was used for comparison of two groups. One way analysis of variance (ANOVA) followed by Tukey test was applied for multiple comparisons. The differences were considered to be significant when the *P* value was less than 5%.

## Results

### MeHg Upregulates IL-6 Expression in Astrocytes

**Figure 1:**
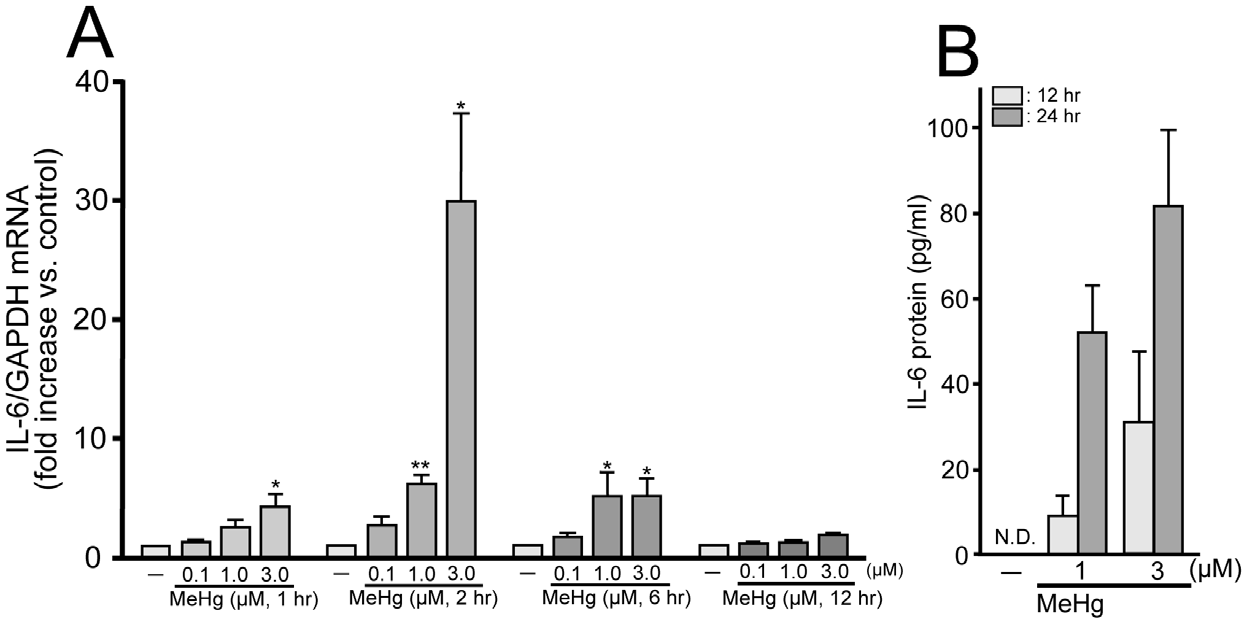
MeHg-induced IL-6 mRNA upregulation and protein release from astrocytes. (**A**) Effect of MeHg on IL-6 mRNA expression in astrocytes. MeHg (1–3 µM) transiently increased IL-6 expression and the induction peak was observed at 2 hr exposure. Low concentration of MeHg (0.1 µM) had no effect on IL-6 expression. *P<0.05 and **P<0.01 vs. control. (**B**) MeHg-induced IL-6 protein production to the supernatant from astrocytes. MeHg (1 or 3 µM, 12 or 24 hr) induced IL-6 production. The 12-hr exposure exhibited a lower level of IL-6 release than that with 24-hr exposure of MeHg.

We first performed transcriptome analysis in cultured astrocytes stimulated with MeHg (10 µM) using DNA microarray (Table 1). MeHg changed the expressions of a large number of genes including those of cytokines and chaperones in astrocytes. Among them, interleukin-6 (IL-6) mRNA showed the most remarkable increase (638 fold), and we confirmed its upregulation using quantitative RT-PCR. The increase in IL-6 mRNA expression was concentration-dependent over a concentration range of from 0.1 to 3 µM with 2 hr-exposure (0.1 µM, 2.6±0.7; 1.0 µM, 6.1±0.7; 3.0 µM, 30.0±7.2 fold increase vs. control, n = 3) (Fig. 1A). The low concentration of MeHg (0.1 µM) never increased IL-6 mRNA expression at any exposure time tested (1–12 hr) (1 hr, 1.3±0.1; 2 hr, 2.6±0.7; 6 hr, 1.7±0.2; 12 hr, 1.2±0.2 fold increase vs. control, n = 3). The increase in the IL-6 mRNA level was transient and reached the maximal level at 2 hr after the exposure with 1 µM (1 hr, 2.5±0.6; 2 hr, 6.1±0.7; 6 hr, 5.1±2.0; 12 hr, 1.2±0.1 fold increase vs. control, n = 3) and 3 µM (1 hr, 4.3±1.1; 2 hr, 29.9±7.2; 6 hr, 5.1±1.6; 12 hr, 1.9±0.2 fold increase vs. control, n = 3) of MeHg. ELISA analysis of the supernatants showed that MeHg (1 and 3 µM, 24 hr) increased IL-6 derived from astrocytes (1 µM MeHg, 51.5±11.4 pg/ml; 3 µM MeHg, 80.9±18.6 pg/ml, n = 4) (Fig. 1B). With 12-hr exposure, a lower level of IL-6 release was observed (1 µM MeHg, 8.7±5.0 pg/ml; 3 µM MeHg, 30.5±16.9 pg/ml, n = 4). Without MeHg stimulation, no detectable level of IL-6 was observed (n.d.).

**Table 1:** A list of top 5 genes upregulated in astrocytes by MeHg (10 µM, 2 hr).

gene ID	gene name	fold increase
**M26744**	interleukin-6 (IL-6)	638
**Z27118**	heat shock protein 70 (Hsp70)	98
**S67722**	cyclooxygenase-2 (COX-2)	35
**X06769**	c-fos	24
**M28259**	fibronectin	18

### Activation of P2Y_1_ Receptor and Subsequent p38 Phosphorylation Mediate IL-6 Expression in Astrocytes

**Figure 2:**
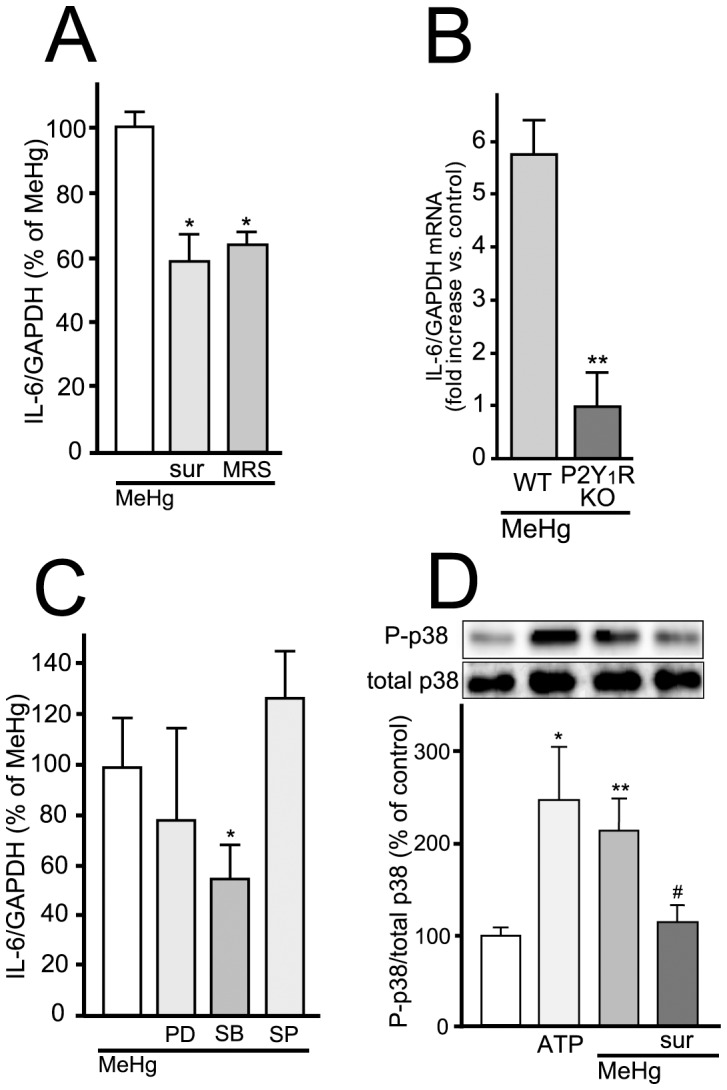
IL-6 upregulation by MeHg is mediated by P2Y_1_ receptors followed by p38 activation. (**A**) P2Y_1_ receptor blockade suppresses IL-6 mRNA expression induced by MeHg. MeHg (3 µM, 2 hr)-increased IL-6 mRNA expression was inhibited by either suramin (sur, 100 µM) or MRS2179 (MRS, 10 µM). *P<0.05 vs. MeHg. (**B**) P2Y_1_ receptor mediates MeHg-induced IL-6 mRNA expression. P2Y_1_R KO astrocytes exhibited no increase in IL-6 mRNA with MeHg (3 µM, 2 hr). **P<0.01 vs. WT. (**C**) Contribution of p38 in MeHg-induced IL-6 mRNA expression. The IL-6 mRNA expression evoked by MeHg (3 µM, 2 hr) was inhibited by SB203580 (SB, 10 µM) but not by PD98059 (PD, 10 µM) or SP600125 (SP, 10 µM). *P<0.05 vs. MeHg. (**D**) Downstream signaling molecule of P2Y_1_ receptor is p38. MeHg (3 µM, 30 min)-induced p38 phosphorylation was inhibited by suramin (sur, 100 µM). ATP (100 µM) also induced p38 phosphorylation. *P<0.05, **P<0.01 vs. control, ^#^P<0.05 vs. MeHg.

The mechanisms underlying the MeHg-evoked IL-6 production were investigated. Since astrocytes release or leak ATP that functions as a gliotransmitter or inflammatory mediator in response to various environmental changes, we firstly focused on ATP/P2 receptor-mediated signals. The MeHg (3 µM, 2 hr)-evoked increase in IL-6 mRNA was significantly suppressed by the broad P2 receptor antagonist suramin (100 µM) (MeHg/suramin, 58.7±8.1% of MeHg, n = 8) (Fig. 2A). The selective P2Y_1_ receptor antagonist MRS2179 (10 µM) also inhibited the IL-6 mRNA upregulation to a similar extent (MeHg/MRS2179, 64.0±4.0% of MeHg, n = 9), suggesting the predominant involvement of P2Y_1_ receptors in the MeHg-evoked IL-6 production. In addition, MeHg (3 µM, 2 hr) failed to increase IL-6 mRNA in P2Y_1_R KO astrocytes (WT, 5.8±0.7; P2Y_1_R KO, 1.0±0.7 fold increase vs. control, n = 3) (Fig. 2B), confirming that P2Y_1_ receptors are necessary in this process. As for intracellular signaling mechanisms, it is known that ATP activates mitogen-activated protein kinases (MAPKs) including ERK1/2, JNK, and p38 in glial cells via several types of P2 receptors [24], [25], [26], [27]. PD98059 (10 µM), an inhibitor of mitogen-activated protein kinase kinase, the upstream activator of ERK [28] and SP600125 (10 µM), an inhibitor of SAPK/JNK [29], exhibited no effect on the IL-6 expression (MeHg/PD98059, 78.3±37.5% of MeHg, n = 4; MeHg/SP600125, 127.5±19.5% of MeHg, n = 6) (Fig. 2C). In contrast, a p38 inhibitor, SB203580 (10 µM) [30], significantly suppressed the MeHg-mediated increase in IL-6 mRNA (MeHg/SB203580, 54.7±13.9% of MeHg, n = 6). Western blotting analysis revealed that MeHg (3 µM, 30 min) induced p38 phosphorylation, which was strongly blocked by suramin (control, 100.0±8.5; MeHg, 210.0±36.3; MeHg/suramin, 114.0±19.7% of control, n = 4) (Fig. 2D). The phosphorylation of p38 in astrocytes was mimicked by ATP (100 µM, 30 min) (246.3±58.0% of control, n = 4). Overall, the data suggest that the activation of P2Y_1_ receptors and subsequent phosphorylation of p38 MAPK pathway should be involved in the MeHg-evoked IL-6 production.

### MeHg Evoked Release of ATP in Astrocytes

**Figure 3:**
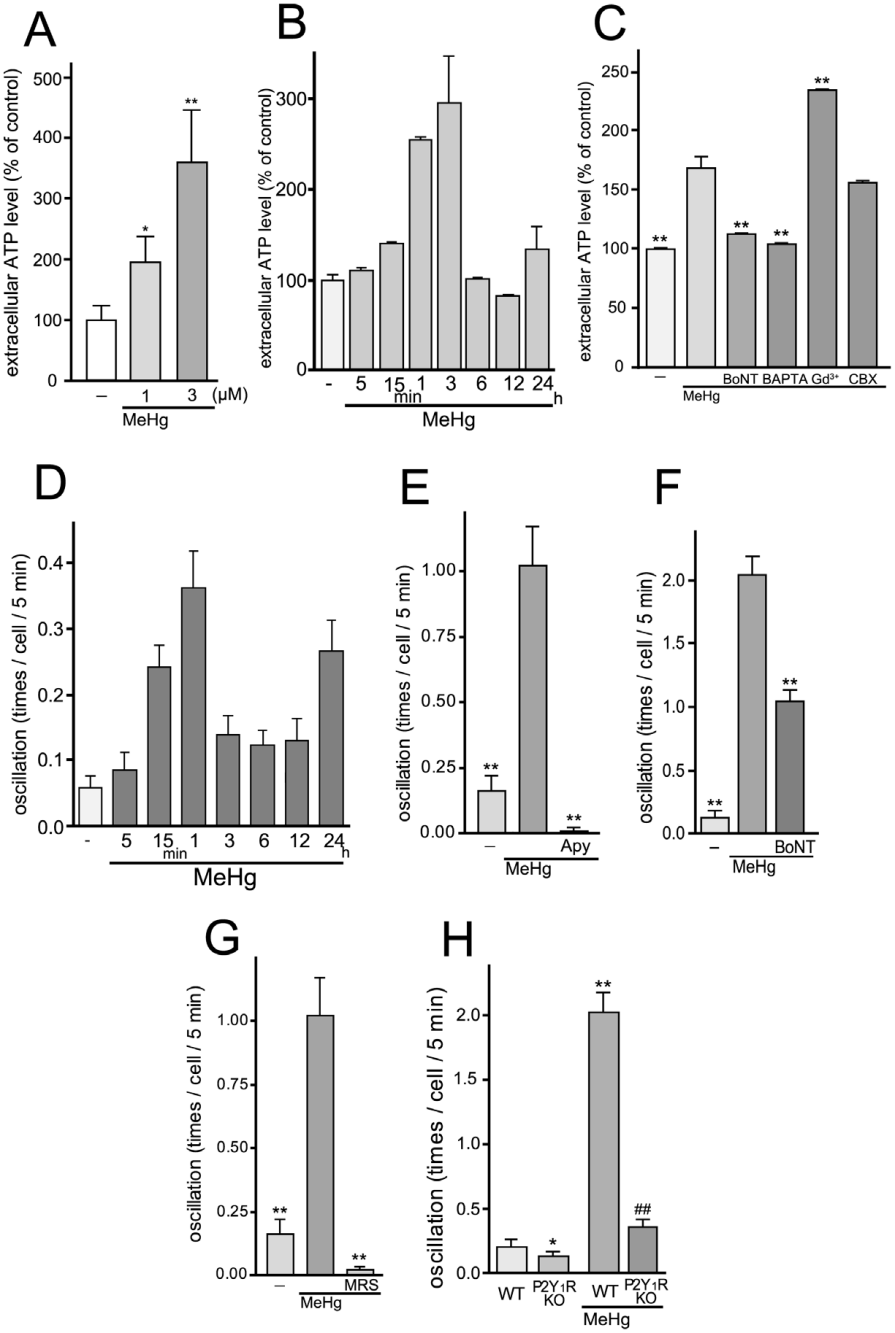
Exocytotic ATP release and Ca^2+^ oscillation in astrocytes evoked by MeHg. (**A**) MeHg increased the extracellular ATP level of astrocytes. The effect of MeHg on the ATP release was concentration-dependent. MeHg was applied to the cell 15 min before the ATP measurement. *P<0.05 and **P<0.01 vs. control. (**B**) The time-course of extracellular ATP level of MeHg-treated astrocytes. MeHg (3 µM) increased extracellular ATP level of astrocytes, which was transient and peaked at 3 hr. (**C**) MeHg induces exocytotic ATP release from astrocytes. BoNT (5 units/ml, 24 hr pretreatment) and BAPTA-AM (10 µM) abolished MeHg (3 µM, 15 min)-induced ATP release from astrocytes. Gd^3+^(50 µM) rather enhanced ATP release, and CBX (100 µM) had no effect. **P<0.01 vs. MeHg. (**D**) The time-course of MeHg-induced changes in the frequency of Ca^2+^ oscillation. MeHg increased frequency of Ca^2+^ oscillation in astrocytes, which was transient and peaked at 1 hr. (**E**) MeHg increases the frequency of spontaneous calcium oscillations in astrocytes via enhancing ATP release. The number of Ca^2+^ oscillations was significantly increased by MeHg (3 µM, 30 min) and the increase was abolished by apyrase (Apy, 20 units/ml). **P<0.01 vs. MeHg. (**F**) Exocytotic pathway contributes to the MeHg-increased Ca^2+^ oscillation. BoNT (5 units/ml, 24 hr pretreatment) reduced the number of Ca^2+^ oscillations. **P<0.01 vs. MeHg. (**G**) Inhibitory effect of P2Y_1_ receptor antagonist on the Ca^2+^ oscillation frequency increased by MeHg. Ca^2+^ oscillation frequency was dramatically increased with MeHg (3 µM), and the increase was blocked by MRS2179 (MRS, 10 µM). **P<0.01 vs. MeHg. (**H**) P2Y_1_R is essential for the Ca^2+^ oscillation evoked by MeHg. WT astrocytes exhibited enhanced Ca^2+^ oscillation by MeHg (3 µM) but not in P2Y_1_R KO astrocytes. *P<0.05, **P<0.01 vs. control (WT), ^##^P<0.01 vs. MeHg (WT).

Next we examined whether MeHg elicits the release of ATP from astrocytes. To measure the release of ATP, we used a luciferin-luciferase based chemiluminescence assay. MeHg (1 and 3 µM for 15 min) increased the extracellular ATP level to 194.6±43.2% (1 µM MeHg) and 358.5±87.1 (3 µM MeHg) % of the pre-stimulated basal control level (77.0±18.0 pM; control, 100±22.9%, n = 10) (Fig. 3A). We analyzed the MeHg (3 µM)-evoked time-course of ATP release and found that it was transient, i.e., it was initiated at 15 min and peaked at 1 to 3 hr, and then was back to non-stimulated basal level in 6 hr (basal ATP level, 0.50±0.03 nM; control, 100.0±5.6%; 5 min, 110±3.2%; 15 min, 140.1±1.0%; 1 hr, 255.1±2.4%; 3 hr, 296.0±51.6%; 6 hr, 101.6±0.9%; 12 hr, 82.3±0.6%; 24 hr, 134.14±25.5%, n = 5) (Fig. 3B). It has been reported that ATP is released from astrocytes via several pathways including exocytosis and diffusion through ATP-permeable plasma membrane channels such as connexin hemichannels, pannexin channels, Maxi-anion channels or P2X_7_ receptors [31], [32], [33], [34], [35], [36], [37]. Using several pharmacological inhibitors, we found that neither Gd^3+^ (50 µM, a maxi-anion channel blocker) [33], nor carbenoxolone (CBX, 100 µM, an inhibitor of connexin hemi-channel, pannexin channel and P2X_7_ receptor) [36], [38], [39] showed an inhibitory effect on the MeHg (3 µM, 15 min)-evoked ATP release from astrocytes (MeHg, 169.0±9.6%; MeHg/Gd^3+^, 234.8±1.0%; MeHg/CBX, 156.3±1.9% of control, n = 4) (Fig. 3C). In contrast to these inhibitors, both Botulinum toxin A (BoNT) (5 units/ml, 24 hr pretreatment), a toxin that cleaves SNAPs [40], [41], thereby preventing exocytosis, and the intracellular Ca^2+^ chelator BAPTA-AM (10 µM) significantly suppressed the MeHg-induced ATP release from astrocytes (MeHg/BoNT, 112.8±0.6%; MeHg/BAPTA, 104.0±0.5% of control, n = 4) (Fig. 3C).

We also evaluated the release of ATP using another indicator, i.e., Ca^2+^ oscillation in astrocytes, because released ATP auto-stimulates P2 receptors to increase the frequency of Ca^2+^ oscillations or increase the distance of Ca^2+^ waves in astrocytes [20], [42], [43]. The time-course of changes in frequency of Ca^2+^ oscillation was also transient. After the addition of MeHg (3 µM), the frequency of spontaneous Ca^2+^ oscillations increased with time and peaked at 1 hr followed by decrease (control, 0.06±0.02; 5 min, 0.09±0.03; 15 min, 0.24±0.03; 1 hr, 0.36±0.05; 3 hr, 0.14±0.03; 6 hr, 0.12±0.02; 12 hr, 0.13±0.03 times/cell/5 min, n = 40) (Fig. 3D). The MeHg (3 µM, 30 min)-increased Ca^2+^ oscillation was completely suppressed by the nucleotide-degrading enzyme apyrase (20 units/ml) (control, 0.16±0.06 times/cell/5 min; MeHg, 1.02±0.15 times/cell/5 min; apyrase/MeHg, 0.01±0.01 times/cell/5 min, n = 100) (Fig. 3E). Similar to the ATP release, the frequency of Ca^2+^ oscillations was reduced by BoNT (5 units/ml, 24 hr pretreatment) (control, 0.13±0.05 times/cell/5 min; MeHg, 2.04±0.15 times/cell/5 min; MeHg/BoNT, 1.04±0.20 times/cell/5 min, n = 100) (Fig. 3F). Because many reports have shown that the P2Y_1_ receptor is essential in the ATP-mediated astrocytic Ca^2+^ signaling [42], [43], [44], the contribution of P2Y_1_ receptors to the MeHg-evoked Ca^2+^ oscillation was tested using the selective P2Y_1_ receptor antagonist MRS2179 [45] or P2Y_1_R KO mice astrocytes. The MeHg-evoked increase in Ca^2+^ oscillation was significantly suppressed by MRS2179 (10 µM) (MeHg/MRS2179, 0.02±0.01 times/cell/5 min, n = 100) (Fig. 3G) and not observed in astrocytes from P2Y_1_R KO mice (WT/control, 0.21±0.05 times/cell/5 min; P2Y_1_R KO*/*control, 0.13±0.04 times/cell/5 min; WT/MeHg, 2.03±0.15 times/cell/5 min; P2Y_1_R KO*/*MeHg, 0.36±0.06 times/cell/5 min, n = 200) (Fig. 3H). P2Y_1_R KO astrocytes exhibited normal Ca^2+^ responses to exogenously applied UTP (100 µM), a P2Y_2/4_ receptor agonist, but not to the P2Y_1_ agonist 2MeSADP (1 µM) (Fig. S1).

### Astrocyte-derived IL-6 Protects Neurons against MeHg

**Figure 4:**
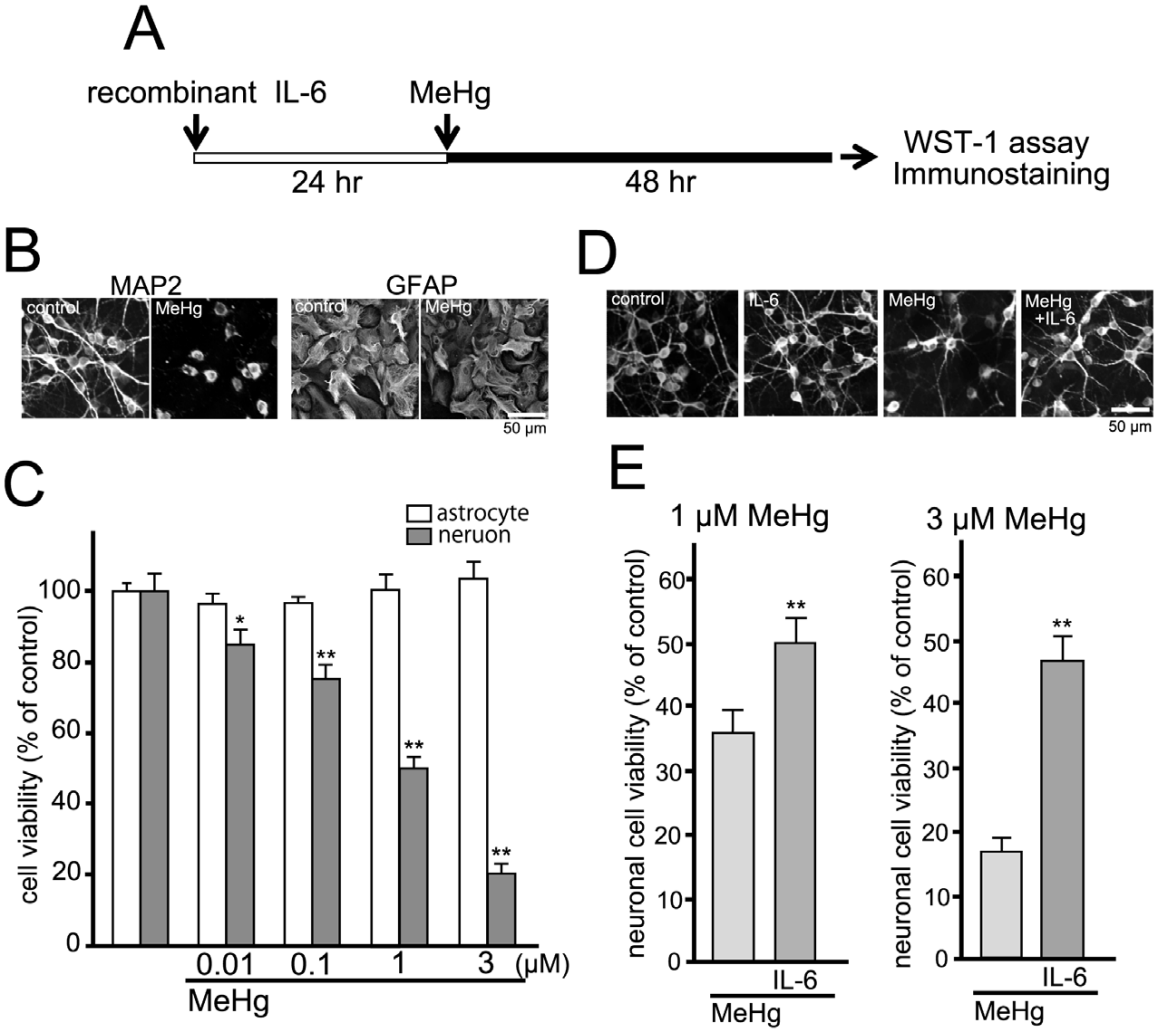
Neuro-protective effect of recombinant IL-6. (**A**) The experimental schedule. Recombinant IL-6 (100 pg/ml) was pretreated to the cell culture 24 hr before MeHg exposure (48 hr). Cells were then fixed for immunostaining or used for WST-1 assay. (**B**) MeHg-induced morphological changes in neurons. MAP2 signals in the processes were disrupted by MeHg (3 µM), whereas the GFAP signals exhibited no differences with or without MeHg. Scale bar: 50 µm. (**C**) MeHg-decreased the neuronal viability. Neuronal viability was significantly reduced by MeHg in a concentration-dependent fashion at a concentration range of from 0.01 to 3 µM (gray columns). MeHg did not affect the astrocytic viability (white columns). *P<0.05, **P<0.01 vs. control. (**D**) Recombinant IL-6 restored the MeHg-induced morphological changes in neurons. The collapsed MAP2 signals in neuronal processes were recovered in the presence of recombinant IL-6 (100 ng/ml). IL-6 did not change the neuronal morphology in controls. Scale bar: 50 µm. (**E**) IL-6 protects neurons against MeHg. Recombinant IL-6 (100 ng/ml) significantly restored the MeHg (1 or 3 µM)-reduced neuronal viability. **P<0.01 vs. MeHg.

We [18] and others [19], [46], [47], [48], [49], [50], [51], [52] have already reported that IL-6 had neuro-protective effects against several types of insults. Using immunocytochemical analysis and WST-1 assay, we evaluated whether IL-6 showed neuro-protection against MeHg. As shown in Fig. 4B, healthy cortical neurons exhibited clear cell bodies and extended dendrites when stained with anti-MAP2 antibody (Fig. 4B, MAP2-control). After MeHg treatment (3 µM, 48 hr), the anti-MAP2 signals were dramatically changed into signals with only cell body-like structures and fragmented or bead-like process structures (Fig. 4B, MAP2-MeHg). In contrast to neurons, anti-GFAP signals exhibited no significant changes with or without MeHg (Fig. 4B, GFAP). WST-1 assay revealed that MeHg (48 hr) decreased neuronal viability in a concentration-dependent manner over a range of from 0.01 to 3 µM (control, 100±5.0%; 0.01 µM, 84.9±4.3%; 0.1 µM, 75.2±4.3%; 1 µM, 50.0±3.2%; 3 µM, 19.9±3.2%) (Fig. 4C, gray columns), whereas astrocytes showed no decrease in cell viability (control, 100±2.4%; 0.01 µM, 96.6±2.9%; 0.1 µM, 96.7±1.8%; 1 µM, 100.6±4.1%; 3 µM, 103.9±4.6%) (Fig. 4C, white columns). Recombinant IL-6 protein (100 pg/ml, 24 hr pretreatment) suppressed the MeHg-induced morphological changes in neurons (Fig. 4D, MeHg+IL-6). IL-6 itself had no significant morphological effect on neurons (Fig. 4D, IL-6). Recombinant IL-6 protein also restored the MeHg (1 or 3 µM, 48 hr)-reduced neuronal viability (MeHg 1 µM, 35.5±3.9%; MeHg 3 µM, 16.4±2.4%; MeHg 1 µM/IL-6, 49.7±4.2%; MeHg 3 µM/IL-6, 46.7±4.0%, n = 20) (Fig. 4E). IL-6 itself had no significant effect on neuronal cell viability (93.2±3.3%, n = 14). When IL-6 was added to the neuronal culture 12 hr after MeHg treatment, it did not show neuro-protection (data not shown). As it is known that neuronal culture contains small portion of astrocytes, we estimated glial contamination in the culture of cortical neurons by immunocytochemical analysis. In our culture condition, almost all cells were positive to MAP2 (98.8±1.2%, n = 780), and remained 1.2±0.2% of cells were positive to GFAP, suggesting that glial contamination could be negligible.

**Figure 5:**
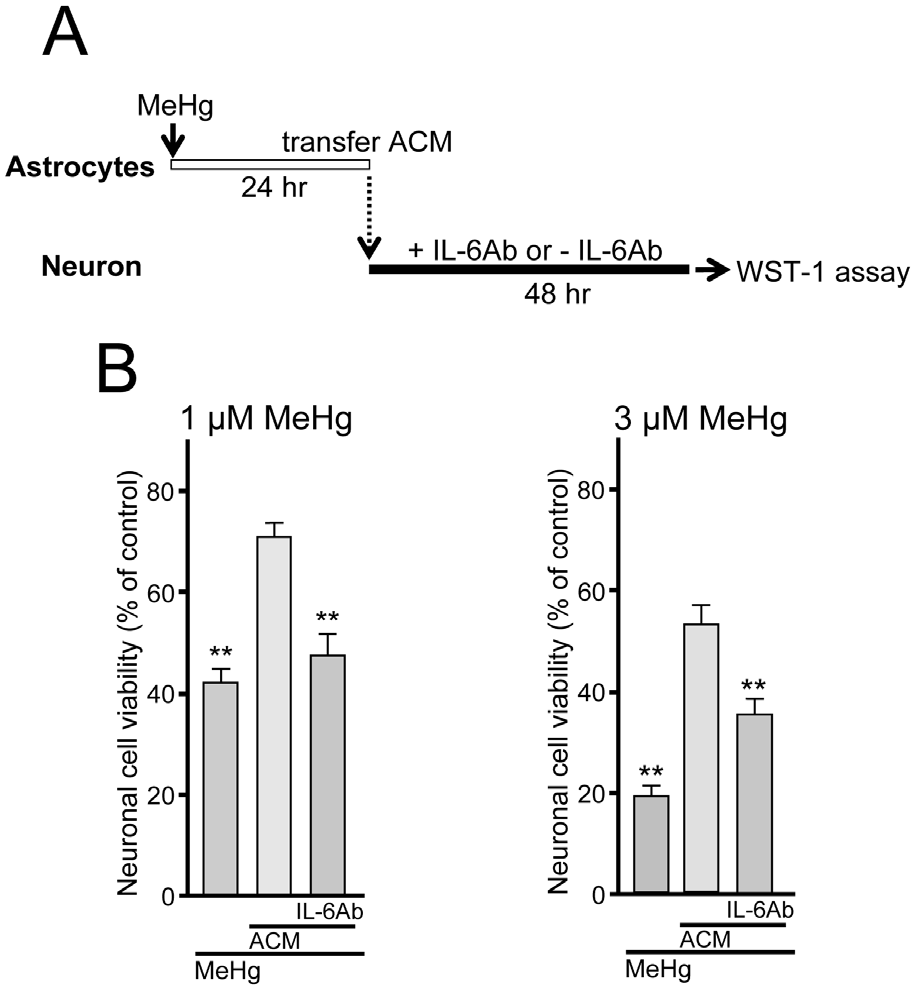
Neuro-protection by astrocyte-derived IL-6. (**A**) The experimental schedule. MeHg (1 or 3 µM) was treated to the astrocyte culture for 24 h and the ACM was transferred into the neuronal culture. The ACM-treated neuronal cultures were further incubated for 48 h with or without IL-6 antibody. (**B**) ACM restored the reduction in neuronal viability evoked by MeHg (1 or 3 µM, 48 hr). The ACM-induced neuro-protection against MeHg was significantly reduced by IL-6 antibody (100 ng/ml). **P<0.01 vs. MeHg.

We then examined the neuro-protective effect of astrocyte-conditioned medium (ACM, MeHg-treated astrocytes conditioned medium). As shown in figure 5A, astrocytes were incubated with MeHg (1 or 3 µM, 24 hr) and ACM was transferred into neuronal culture and neurons were further incubated in the either presence or absence of anti-IL-6 antibody (100 ng/ml) for 48 hr. ACM restored the MeHg-decreased neuronal viability (1 µM MeHg, 41.8±3.0%; 1 µM MeHg/ACM, 70.6±2.9%; 3 µM MeHg, 18.9±2.0%; 3 µM MeHg/ACM, 52.3±4.0%, n = 30) (Fig. 5B). The protective effects of ACM were abolished by IL-6 antibody (1 µM MeHg/ACM/IL-6 Ab, 47.4±4.3%; 3 µM MeHg/ACM/IL-6 Ab, 35.5±3.1%, n = 15), indicating that the ACM-mediated neuro-protection is dependent on IL-6. IL-6 antibody itself showed no direct effect on neuronal cell viability (97.7±9.8%; n = 5). When IL-6 was added to neurons 12 hr after MeHg, it no longer showed neuro-protection (1 µM of MeHg, 57.0±6.4%; MeHg/IL-6, 64.6±8.0%, n = 6, P>0.05), suggesting that an effective time window seems to be present for the IL-6- or ACM-mediated neuro-protection.

### Neuro-protective Action of IL-6 is Mediated by Neuronal A_1_ Receptor

**Figure 6:**
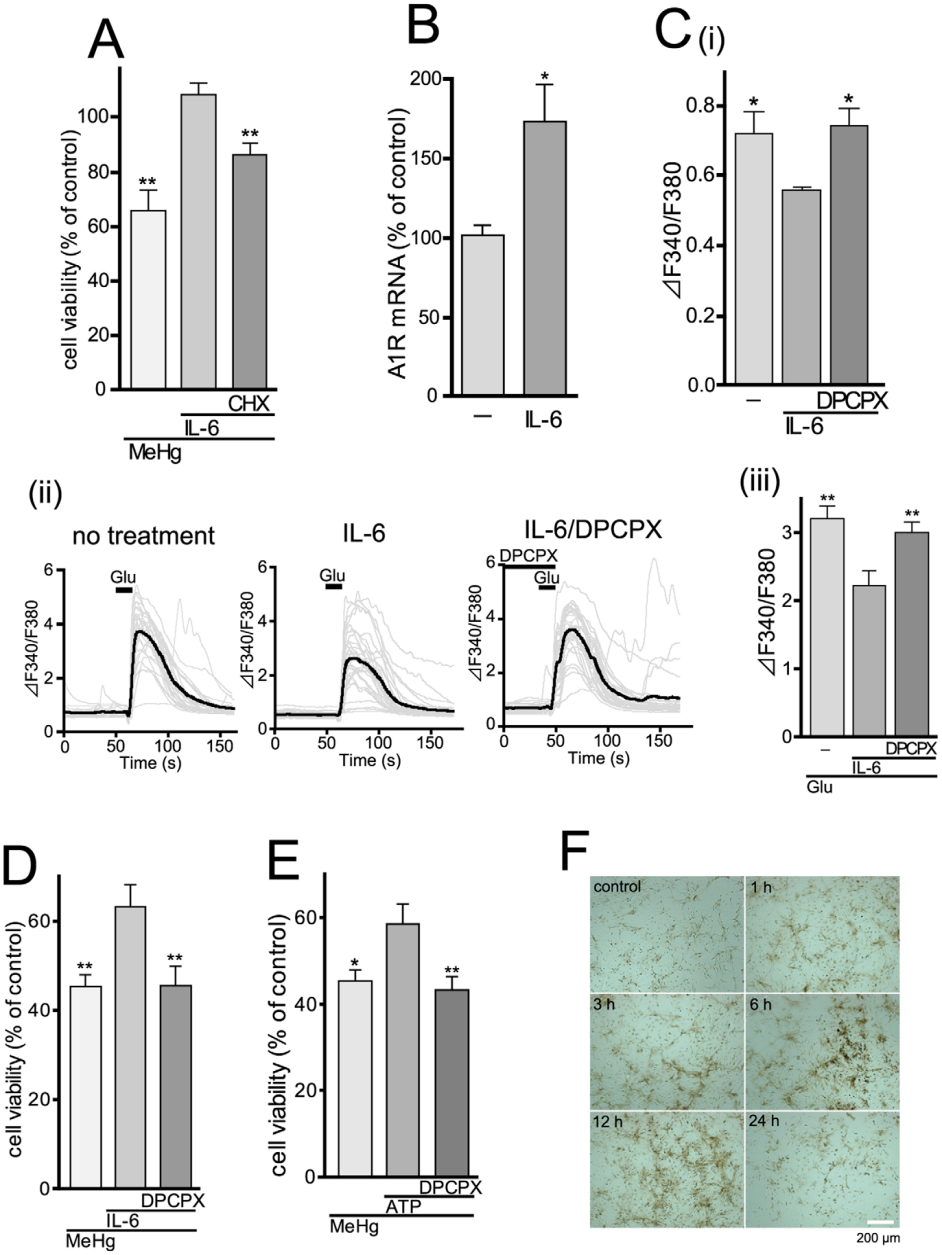
Adenosine A_1_ receptor mediates neuro-protection against MeHg via suppressing excitatory neurotransmission. (**A**) Newly synthesized proteins participate in the IL-6-mediated neuro-protection by recombinant IL-6 (100 pg/ml) was suppressed by CHX (1 µM). **P<0.01 vs. MeHg/IL-6. (**B**) Upregulation of adenosine A_1_ receptor mRNA by recombinant IL-6 (200 pg/ml, 2 hr) in the cortical neurons. *P<0.05 vs. no treatment. (**C**) Changes in [Ca^2+^]i in control and IL-6-treated cortical neurons, showing effect of A_1_ receptors. (i) The basal [Ca^2+^]i level in IL-6-treated (100 pg/ml, 24 hr) neurons was significantly lower than that in control neurons. This decrease was restored by DPCPX (1 µM). *P<0.05 vs. IL-6 alone. (ii) Representative traces of the glutamate-evoked increases in [Ca^2+^]i in non-treated control (left), IL-6-treated (100 pg/ml, 24 hr) (right) and IL-6-treated neurons in the presence of 1 µM DPCPX. Glutamate (10 µM) was added to the neurons for 10 s. Bold line in each panel showed averaged changes in [Ca^2+^]i in neurons, which was summarized in (iii). The glutamate-evoked increase in [Ca^2+^]i in IL-6-treated neurons was significantly lower than that in control neurons, which was restored by DPCPX. **P<0.01 vs. Glu/IL-6. (**D**) A_1_ receptor-mediated neuro-protection by IL-6. The protective effect of IL-6 (100 pg/ml) was suppressed by DPCPX (1 µM). **P<0.01 vs. MeHg/IL-6. (**E**) ATP-induced neuro-protection is mediated by A_1_ receptor. Exogenously applied ATP (100 µM) restored the MeHg (1 µM, 48 hr)-reduced neuronal viability, and this effect was blocked by DPCPX (1 µM). *P<0.05, **P<0.01 vs. MeHg/ATP. (**F**) The MeHg-evoked increase in activity of ecto-ATPases in astrocytes. Activity of ecto-ATPases was analyzed by an enzyme histochemical assay. When stimulated with MeHg (3 µM), the activity (shown as brown signals) was increased, which peaked at around 6 to 12 hr. Scale bar, 200 µm.

The neuro-protection by recombinant IL-6 was significantly inhibited by 1 µM cycloheximide (CHX), an inhibitor of protein synthesis (MeHg, 65.7±7.6%; MeHg/IL-6, 108.3±4.5%; MeHg/IL-6/CHX, 86.1±4.2%, n = 5) (Fig. 6A). CHX (1 µM, added to the culture 24 hr after IL-6 treatment) alone had no effect on neuronal cell viability (107.2±2.2%, n = 5). Thus, it appears that IL-6 would newly synthesize neuro-protective molecules that would account for the neuro-protection against MeHg. IL-6 has been reported to increase A_1_ receptor expression thereby inducing neuro-protection against cytotoxicity [53], [54]. We found that recombinant IL-6 (200 pg/ml, 2 hr) significantly increased adenosine A_1_ receptor mRNA in cortical neurons (control, 100±6.7%; IL-6, 173.1±23.9%, n = 7) (Fig. 6B). We further tested whether the IL-6-mediated A_1_ receptor upregulation contributes to excitability of cortical neurons. Firstly, we investigated whether pre-treatment with IL-6 might affect basal level of [Ca^2+^]i in the cortical neurons, because this can reflect a baseline activity of synaptic transmission. Thus, when excitatory synaptic transmission is inhibited by either TTX, antagonists of glutamate receptors, or agonists of presynaptic auto-receptors such as A_1_ receptors, the basal [Ca^2+^]i is decreased [20], [21]. As shown in Fig. 6C (i), the basal [Ca^2+^]i in IL-6-treated neurons (100 pg/ml, 24 hr) was lower than that in control neurons, which was restored by an A_1_ receptor antagonist DPCPX (1 µM, 5 min) (F340/F380: no treatment, 0.72±0.06; IL-6, 0.56±0.01; IL-6/DPCPX, 0.74±0.05, n = 99). Secondly, we tested whether the glutamate-evoked responses were affected by the IL-6-treatment. The glutamate (10 µM)-evoked increase in [Ca^2+^]i in IL-6-treated neurons was smaller than that in control neurons(F340/F380: no treatment, 3.2±0.2; IL-6, 2.2±0.2, n = 99), which was also restored by 1 µM DPCPX (IL-6/DPCPX, 3.0±0.2, n = 99) (Fig. 6C (ii) (iii)). DPCPX alone (1 µM) never affected the neuronal viability (control, 100.0±2.9%; DPCPX, 95.6±6.0%, n = 5). All these findings suggest that IL-6 could regulate excitability of cortical neurons by increasing both expression and function of adenosine A_1_ receptors, which might contribute to neuro-protection against MeHg.

Next we asked whether the increased A_1_ receptors in cortical neurons are related to neuro-protection against MeHg. The protective effect of IL-6 (100 pg/ml, 24 hr) against MeHg (1 µM) was abolished by DPCPX (1 µM) (MeHg, 45.3±2.6%; MeHg/IL-6, 63.1±4.9%; MeHg/IL-6/DPCPX, 45.4±4.5%, n = 18) (Fig. 6D). Similarly, simultaneous application of ATP (100 µM) with MeHg also exhibited a neuro-protective effect, which was blocked by DPCPX (1 µM) (MeHg, 45.3±2.6%; MeHg/ATP, 58.5±4.7%; MeHg/ATP/DPCPX, 43.2±3.1%, n = 18) (Fig. 6E). As tonic A_1_ receptor activation would require an increased level of extracellular adenosine, we then performed an enzyme histochemistry for evaluating activity of ecto-ATPases in astrocytes. As shown in figure 6F, MeHg (3 µM) increased the activity of ecto-ATPases (shown as brown signals), which peaked around 6–12 hr. The MeHg-evoked increase in extracellular ATP was observed prior to the increase in ecto-ATPases activity (i.e. peaked at 3 hr) (Fig. 3B). These findings strongly suggest that the MeHg-treated ACM should contain higher levels of adenosine.

## Discussion

MeHg easily passes the blood-brain barrier and causes serious damage in the CNS. Unlike neurons, its effect on glial cells has received only limited attention. In the present study, we demonstrated that, when exposed to MeHg, astrocytes exhibited neuro-protection against MeHg, in which ATP/P2Y_1_ receptor-mediated signals and subsequent IL-6 production in astrocytes have a pivotal role.

MeHg significantly increased extracellular ATP level of astrocytes. Although it has been described that MeHg induces astrocytic swelling [55], [56], we did not observe significant morphological changes or injured structures in astrocytes when evaluated by immunocytochemical analysis using anti-GFAP antibody. The increase in the extracellular ATP level did not seem to be due to leakage by cell damage because MeHg never decreased the astrocyte viability (Fig. 4). Although astrocytes release ATP by multiple mechanisms, exocytosis might be one of them because both BoNT and BAPTA-AM reduced the ATP release, while neither CBX nor Gd^3+^ inhibited the release (Fig. 3). However, the finding that the inhibition of the Ca^2+^ oscillation by BoNT was incomplete suggests that SNARE-independent pathways for ATP release might also be involved (Fig. 3F). With regard to ATP exocytosis, a recent report has demonstrated that lysosomes mediate at least part of the exocytotic ATP release in astrocytes [37]. In addition to lysosomes, vesicular nucleotide transporter (VNUT) has been reported to mediate exocytotic ATP release [57]. We must await further studies to clarify the involvement of lysosomes and/or VNUT-vesicles in the MeHg-evoked ATP release by astrocytes or the initial target molecule(s) of MeHg. However, our present data clearly showed that, when astrocytes sense MeHg, they release ATP in part by exocytosis. This initial ATP release should be a key response because subsequent events, e.g., IL-6 production or neuro-protection by astrocytes, were dependent on ATP/P2Y_1_ receptors.

In the transcriptome analysis, we found that MeHg upregulated several genes, among which IL-6 mRNA showed the most remarkable increase (table 1). The MeHg-evoked upregulation of IL-6 mRNA peaked at 2 hr and IL-6 protein production was observed at 12 or 24 hr (Fig. 1A and 1B). Such time lag between IL-6 mRNA and protein expression can be also seen in other reports of various stimuli-induced IL-6 expression/production in astrocytes [58], [59], [60], [61]. It would take longer time for the *de novo* synthesis or release of IL-6 after its mRNA upregulation in astrocytes, but we must await further investigation to clarify it since we did not check the IL-6 release at earlier (<12 hr) or later time points (>24 hr). The MeHg-evoked IL-6 production was dependent on the activation of P2Y_1_ receptors in astrocytes, because the IL-6 production was (i) reduced by the antagonists to P2Y_1_ receptors, (ii) was not observed in P2Y_1_R KO astrocytes, and (iii) was mimicked by exogenously applied ATP. As for the downstream signaling of P2Y_1_ receptors, we focused on MAPKs. Of the three MAPK members (i.e. ERK1/2, JNK, and p38), only the p38 inhibitor SB203580 suppressed the MeHg-induced IL-6 mRNA upregulation (Fig. 2C). In addition, the phosphorylation of p38 by MeHg was blocked by the P2 receptor antagonist. ATP itself also evoked p38 phosphorylation (Fig. 2D). All these findings suggest that MeHg triggered the exocytosis of ATP, which in turn autostimulates P2Y_1_ receptors, thereby leading to p38-mediated IL-6 production in astrocytes. Many reports have shown that p38 is required for the induction of IL-6 in astrocytes, together with a variety of stimuli including prostaglandin E_2_ [62], co-stimulation with IL-6 and IL-17 [60], thromboxane A_2_ [63], oncostatin M [64], and ICAM-1 ligation [65]. The p38 activation might be a common key pathway for IL-6 expression in astrocytes. The p38 inhibitor and P2 receptor antagonists (i.e. suramin and MRS2179) exhibited lesser extent of inhibitory effects than those by P2Y_1_R KO astrocytes. This discrepancy might be due to their lower concentrations because previous studies have shown that 10 µM of SB203580 does not show complete blockade for stimuli-induced p38 phosphorylation in astrocytes [66], [67], [68], [69], [70]. Similarly, we previously showed that neither suramin (100 µM) nor MRS2179 (1 µM) completely suppressed the ATP (100 µM)-evoked Ca^2+^ transient (i.e. about 70% suppression) [26].

Since IL-6 is a cytokine with major regulating effects on the inflammatory response, in general an elevation in the proinflammatory cytokine IL-6 is considered to have a damaging effect on neurons. We and others, however, reported that IL-6 could protect neurons against a variety of damages including trauma, ischemia, excitotoxicity and oxidative stress [18], [19], [46], [47], [48], [49], [50], [51], [52]. In the present study, astrocytes showed neuro-protection against MeHg via IL-6-mediated mechanisms because ACM-induced neuro-protection was IL-6 dependent (Fig. 5) and exogenously applied recombinant IL-6 protein mimicked the neuro-protection (Fig. 4). One possible mechanism of the IL-6-mediated neuro-protection is that IL-6 stimulates the induction of neuro-protective molecules. We showed that the neuro-protection by IL-6 disappeared in the presence of a protein synthesis inhibitor CHX (Fig. 6A), suggesting that *de novo* synthesis of certain neuro-protective molecules appears to be required. Recent reports by Biber et al. demonstrated that IL-6 inhibits the glutamate-induced excitototoxicity of cortical neurons requires adenosine A_1_ receptor functions in neurons [53], [54]. The IL-6 increased both mRNA and proteins of adenosine A_1_ receptors in the neurons, and the protection by IL-6 disappeared in the presence of CHX [54]. In the present study, we also found that IL-6 increases A_1_ receptor mRNA expression in cortical neurons (Fig. 6B). IL-6 increased not only mRNA but also A_1_ receptor-mediated tonic inhibition on an excitatory neurotransmitter (Fig. 6C). Supporting these results, the IL-6-induced neuro-protection against MeHg was suppressed by the A_1_ receptor antagonist DPCPX (Fig. 6D). All these findings may support the idea that one of the neuro-protective molecules induced by ACM or IL-6 would be adenosine A_1_ receptors.

However, anti-IL-6 antibody could not abolish the effect of ACM, indicating the involvement of IL-6-independent neuro-protective mechanisms. We considered that the astrocyte-derived ATP itself might function as another neuro-protective molecule because, without ACM, the exogenously applied ATP alone showed neuro-protection (Fig. 6E). Interestingly, this protection by ATP was also inhibited by the antagonist to adenosine A_1_ receptor DPCPX (Fig. 6E). Astrocytic ATP either as ATP [20] or metabolized into adenosine by ecto-nucleotidases [71], [72], inhibits excess excitatory synaptic transmission, leading to inhibition of excitatory neuronal death. Our time-lapse analysis of extracellular ATP level and enzyme histochemistry have shown that MeHg gradually increased ATP release from astrocytes followed by an increase in ATPase activity (Fig. 3B and 6F). Under this condition, extracellular adenosine would increase and activate neuronal A_1_ receptor.

Since it takes 24 hr for IL-6 to protect neurons against MeHg (Fig. 4), the delayed production of IL-6 (Fig. 1) in astrocytes might be problematic to the neuro-protection, because in general both neurons and astrocytes would be simultaneously exposed to MeHg in situ. However, astrocytes could release ATP in response to MeHg as early as 15 min after MeHg (Fig. 3A), and the released ATP or its metabolite adenosine directly protected neurons against MeHg (Fig. 6), suggesting that astrocytes should show the IL-6-independent neuro-protection even in the early stage. Thus, the astrocyte-mediated neuro-protection shown in the present study could work in situ. Furthermore, we previously showed that activation of P2Y_1_ receptors in astrocytes increased tolerance against oxidative stress by the upregulation of various oxidoreductase genes [26], [73]. Therefore, the astrocytic ATP release and activation of P2Y_1_ receptors appear to be key events that trigger multiple neuro-protective responses.

**Figure 7:**
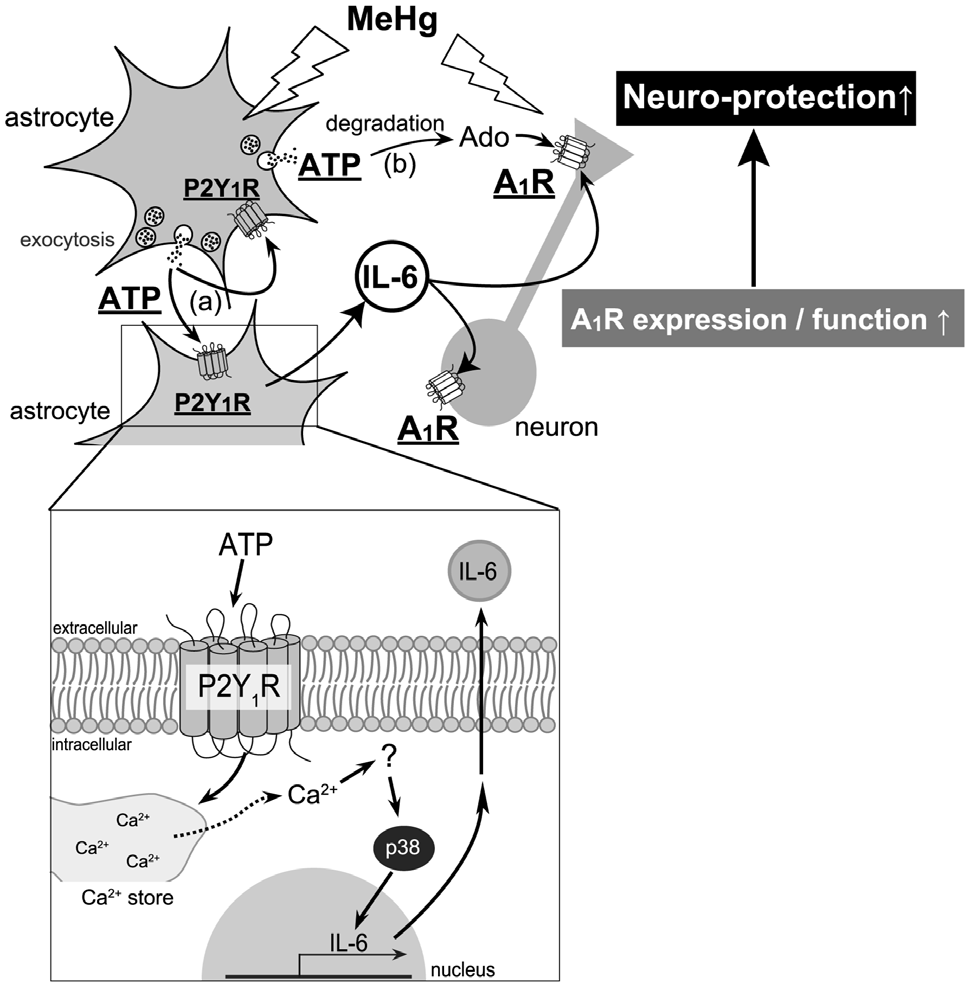
A schematic diagram, illustrating mechanisms underlying astrocyte-mediated neuro-protection against MeHg. MeHg stimulates exocytosis of astrocytic ATP that functions as both (a) autocrine and (b) paracrine signals to reveal neuro-protection, i.e., (a) the released ATP as an autocrine signal, autostimulates P2Y_1_ receptors to induce IL-6 that upregulates neuronal adenosine A_1_ receptors, (b) the released ATP from astrocytes being degraded into adenosine, stimulates neuronal adenosine A_1_ receptors and suppresses neuronal excitability as a paracrine signal, thereby leading to further inhibition of neuronal excitability. As for mechanisms for IL-6 synthesis and release, an increase in [Ca^2+^]i in astrocytes mediated by P2Y_1_ receptors, and subsequent p38 phosphorylation were involved (insert).

Taken together, as summarized in Figure 7, when astrocytes are exposed to MeHg, they exocytose ATP and show a neuro-protective phenotype. The released ATP functions as an autocrine to stimulate P2Y_1_ receptors, thereby leading to the protection of neurons against MeHg via IL-6-mediated pathways. The IL-6 increases neuronal A_1_ receptor expression and function. The released ATP, being metabolized into adenosine, may also function as a paracrine to exert neuro-protection via suppressing excitatory neurotransmission.

## Supporting Information

Figure S1 **Differences in Ca^2+^ responses to 2MeSADP and UTP in WT and P2Y_1_R KO mice.** (**A**) Typical Ca^2+^ responses to the P2Y_1_R agonist 2methyl-thio-ADP (2MeSADP) (1 µM) and the P2Y_2/4_ receptor agonist UTP (100 µM) in control astrocytes obtained from WT mice (upper traces) and those from P2Y_1_R KO mice (lower traces). Although UTP evoked [Ca^2+^]i elevations in both WT and P2Y_1_R KO astrocytes, 2MeSADP failed to produce the [Ca^2+^]i increse in P2Y_1_R KO astrocytes, which was summarized in B.
(TIF)
