# Supplementary figures and images for: Astrocytes Protect Neurons against Methylmercury via ATP/P2Y1 Receptor-Mediated Pathways in Astrocytes
Source: PLoS One. 2013 Feb 28;8(2):e57898. doi: 10.1371/journal.pone.0057898 (PMC3585279; doi:10.1371/journal.pone.0057898)

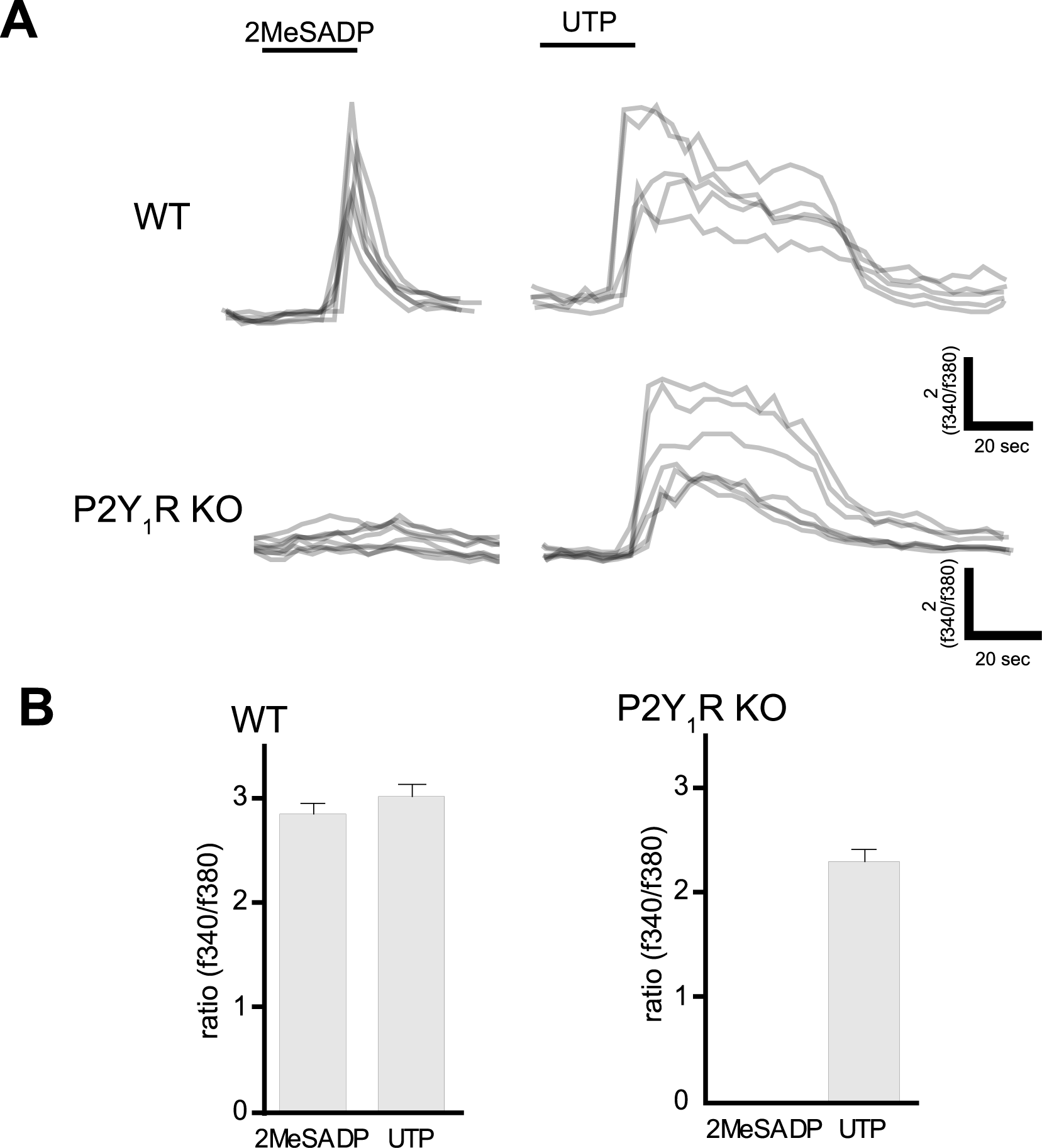

Supplement: Figure S1 — Differences in Ca2+ responses to 2MeSADP and UTP in WT and P2Y1R KO mice. (A) Typical Ca2+ responses to the P2Y1R agonist 2methyl-thio-ADP (2MeSADP) (1 µM) and the P2Y2/4 receptor agonist UTP (100 µM) in control astrocytes obtained from WT mice (upper traces) and those from P2Y1R KO mice (lower traces). Although UTP evoked [Ca2+]i elevations in both WT and P2Y1R KO astrocytes, 2MeSADP failed to produce the [Ca2+]i increse in P2Y1R KO astrocytes, which was summarized in B. (TIF) [file pone.0057898.s001.tif]
